# Reprogramming the Mitochondrion in Atherosclerosis: Targets for Vascular Protection

**DOI:** 10.3390/antiox14121462

**Published:** 2025-12-05

**Authors:** Patrycja Anna Glogowski, Federica Fogacci, Cristina Algieri, Antonia Cugliari, Fabiana Trombetti, Salvatore Nesci, Arrigo Francesco Giuseppe Cicero

**Affiliations:** 1 Department of Veterinary Medical Sciences, University of Bologna, 40064 Ozzano Emilia, Italy; patrycja.glogowski2@unibo.it (P.A.G.); cristina.algieri2@unibo.it (C.A.); antonia.cugliari2@unibo.it (A.C.); fabiana.trombetti@unibo.it (F.T.); salvatore.nesci@unibo.it (S.N.); 2 Cardiovascular Medicine Unit, Heart, Chest and Vascular Department, IRCCS Azienda Ospedaliero—Universitaria di Bologna, 40138 Bologna, Italy; 3 Hypertension and Cardiovascular Risk Research Center, Medical and Surgical Sciences Department, Alma Mater Studiorum—University of Bologna, Sant’ Orsola-Malpighi Hospital, 40138 Bologna, Italy

**Keywords:** mitochondrial reprogramming, atherosclerosis (AS), endothelial dysfunction, vascular protection, mitophagy, vascular smooth muscle cells (VSMC), nanoparticle-based therapies

## Abstract

Cardiovascular diseases (CVDs) remain the leading cause of death worldwide, with a substantial proportion of events occurring prematurely. Atherosclerosis (AS), the central driver of cardiovascular pathology, results from the convergence of metabolic disturbances, vascular inflammation, and organelle dysfunction. Among intracellular organelles, mitochondria have emerged as critical regulators of vascular homeostasis. Beyond their canonical role in adenosine triphosphate (ATP) production, mitochondrial dysfunction—including impaired mitochondrial oxidative phosphorylation (OXPHOS), excessive generation of reactive oxygen species (ROS), accumulation of mitochondrial DNA (mtDNA) damage, dysregulated dynamics, and defective mitophagy—contributes to endothelial dysfunction, vascular smooth muscle cell (VSMC) phenotypic switching, macrophage polarization, and ultimately plaque initiation and destabilization. These insights have established the rationale for mitochondrial “reprogramming”—that is, the restoration of mitochondrial homeostasis through interventions enhancing biogenesis, dynamics, and quality control—as a novel therapeutic paradigm. Interventions that enhance mitochondrial biogenesis, restore mitophagy, and rebalance fission–fusion dynamics are showing promise in preclinical models of vascular injury. A growing array of translational strategies—including small-molecule activators such as resveratrol and Mitoquinone (MitoQ), gene-based therapies, and nanoparticle-mediated drug delivery systems—are under active investigation. This review synthesizes current mechanistic knowledge on mitochondrial dysfunction in ASand critically appraises therapeutic approaches aimed at vascular protection through mitochondrial reprogramming.

## 1. From Residual Risk to Mitochondrial Reprogramming: A New Chapter in Atherosclerosis (AS)

Cardiovascular diseases (CVDs) remain the leading global cause of mortality. According to the Global Burden of Disease (GBD) Study 2023, ischaemic heart disease and stroke continue to rank as the top causes of death worldwide, despite a steady decline in age-standardised mortality rates over the past decades. However, substantial regional and sex-related disparities persist, and premature cardiovascular mortality remains a major global health concern [1]. Atherosclerosis (AS)is a central pathological process underpinning the onset and progression of diverse CVDs. It involves progressive thickening of the arterial wall, driven by subendothelial plaque formation composed of lipids, cholesterol, calcium, fibrin, cellular debris, and metabolic by-products. This remodeling progressively narrows the arterial lumen, potentially leading to complete occlusion and tissue ischemia [2]. Despite major advances with lipid-lowering and anti-inflammatory agents, a substantial residual risk of adverse atherosclerotic events persists [3], underscoring the urgent need for innovative therapeutic strategies. Notably, the pathogenesis of AS is not only shaped by systemic metabolic disturbances but also by intracellular organelle dysfunction, with mitochondria playing a particularly central role [4].

Traditionally, mitochondria have been regarded as the primary source of adenosine triphosphate (ATP) to sustain cellular energy demands. In tissues that depend heavily on mitochondrial oxidative phosphorylation (OXPHOS—such as skeletal myofibers and cardiomyocytes—mitochondria account for ~15% and ~35% of total cell volume, respectively [5,6]. By contrast, vascular endothelial cells (ECs) rely predominantly on anaerobic glycolysis for ATP production, and mitochondria represent only ~2–5% of their cytoplasmic volume in most vascular beds [7]. This comparatively low mitochondrial density led to an underestimation of their role in vascular biology. However, it is now clear that endothelial mitochondria are crucial signaling organelles, particularly through their ability to generate mitochondrial reactive oxygen species (mROS) and regulate calcium homeostasis [8].

Mitochondrial dysfunction in the vascular endothelium is characterized by impaired bioenergetic capacity, excessive mROS production, accumulation of mitochondrial DNA (mtDNA) damage, dysregulated dynamics, and defective mitophagy [9]. These alterations compromise endothelial function, propagate vascular inflammation, and modulate the phenotype and activity of vascular smooth muscle cells (VSMCs) and infiltrating macrophages—ultimately driving atherogenesis and promoting plaque instability [10].

Against this backdrop, the concept of mitochondrial “reprogramming” has emerged as a promising therapeutic paradigm in vascular medicine. In this context, *mitochondrial dysfunction* refers to the maladaptive alterations in bioenergetics, oxidative balance, and organelle turnover that drive vascular injury [11]. Conversely, mitochondrial reprogramming defines the set of compensatory or therapeutic processes—either naturally induced or pharmacologically triggered—that restore mitochondrial quality control, optimize metabolic signaling, and preserve vascular cell homeostasis. Interventions aimed at enhancing mitochondrial biogenesis, notably via peroxisome proliferator-activated receptor-γ coactivator-1α (PGC-1α), restoring quality-control mechanisms such as mitophagy, and rebalancing mitochondrial dynamics by harmonizing fusion and fission processes have demonstrated efficacy in preclinical models of vascular injury [12,13]. A broad range of approaches—including small-molecule activators such as resveratrol and Mitoquinone (MitoQ [14,15], gene-targeted therapies (e.g., PGC-1α overexpression) [16], and advanced drug delivery platforms such as polymeric nanoparticles and liposomes [17]—are currently under active investigation, highlighting the translational potential of this strategy.

This review synthesizes current insights into the role of mitochondrial dysfunction in AS and critically evaluates therapeutic strategies designed to reprogram mitochondrial function for vascular protection. We discuss molecular mechanisms underlying mitochondrial perturbations, review preclinical and clinical evidence for mitochondrial-targeted interventions, and outline future directions poised to accelerate progress in this dynamic field.

## 2. Mitochondrial Dysfunction in Atherosclerosis: From Oxidative Stress to Metabolic Failure

Chronic exposure to atherosclerotic risk factors and adverse lifestyle behaviors induces pathological overproduction of mROS, exhaustion of endogenous antioxidant defenses, and persistent oxidative stress. This imbalance drives peroxidation of lipids, proteins, and nucleic acids, culminating in structural injury, loss of function, and ultimately death in vascular cell types contributing to AS pathophysiology, as summarized in Table 1. Within the vascular wall, oxidized lipoproteins progressively accumulate, fueling plaque development, while oxidative damage to proteins and nucleic acids directly compromises mitochondrial integrity, tricarboxylic acid (TCA) cycle flux, and OXPHOS [18].

### 2.1. Mitochondrial Dysregulation in the Endothelium: Fueling Oxidative Stress, Inflammation, and Atherosclerosis

The vascular endothelium is a continuous monolayer lining the entire cardiovascular system and is indispensable for maintaining vascular homeostasis [19]. In ECs, mitochondria exert functions that extend far beyond ATP production, despite their relatively low abundance. Rather, endothelial mitochondria critically regulate intracellular redox balance, calcium flux, and nitric oxide NO bioavailability—processes that collectively sustain vasodilation, barrier integrity, and anti-inflammatory signaling [20].

Under atherogenic stimuli such as hyperlipidemia, disturbed flow, and chronic inflammation, the mitochondrial electron transport chain (ETC)—particularly complexes I and III—becomes a dominant source mROS [8]. Excessive mROS not only diminishes NO signaling through peroxynitrite formation but also promotes nitrosative stress and oxidative modification of lipids, proteins, and mtDNA [21]. Given its proximity to the ETC and limited repair capacity, mtDNA is highly susceptible to oxidative injury, which impairs transcription of ETC subunits and aggravates respiratory dysfunction. Moreover, upon release into the cytoplasm or extracellular space, damaged mtDNA serves as a potent damage-associated molecular pattern (DAMP), amplifying vascular inflammation [22]. Extramitochondrial mtDNA activates Toll-like receptor 9 (TLR9) and cytosolic DNA sensors such as cyclic guanosine monophosphate-adenosine monophosphate GMP–AMP synthase–stimulator of interferon genes (cGAS–STING), thereby triggering nuclear factor kappa-light-chain-enhancer of activated B cells (NF-κB) signaling and inflammasome assembly [23,24,25].

Mitochondrial dynamics are likewise perturbed under these conditions: atherogenic stimuli favor dynamin-related protein 1 (Drp1)-mediated fission while repressing mitofusin-1 (MFN1) and mitofusin-2 (MFN2)-driven fusion, leading to excessive fragmentation [26,27]. Fragmented mitochondria are more vulnerable to dysfunction and more prone to initiate intrinsic apoptotic signaling [24,28]. At the same time, defective mitophagy—often due to impaired PTEN-induced putative kinase 1 (PINK1) and Parkin RBR E3 ubiquitin ligase (Parkin) signaling—prevents clearance of damaged organelles, perpetuating a self-sustaining cycle of oxidative stress and inflammation [29].

Collectively, these alterations establish a pathophysiological cascade that drives endothelial activation, leukocyte adhesion, increased vascular permeability, and the emergence of a pro-inflammatory, pro-thrombotic endothelial phenotype that underlies the initiation and progression of atherosclerotic lesions [30].

**Table 1:** Mitochondrial dysfunctions in vascular cell types contributing to atherosclerosis pathophysiology.

Cell Type	Mitochondrial Alteration	Key Cellular Mechanisms	Pathological Consequences Atherosclerosis	References
Endothelial Cells (ECs)	- Excess mROS from ETC complexes I and III - mtDNA oxidative damage and release - Imbalanced dynamics: ↑ Drp1-mediated fission, ↓ MFN1/MFN2 fusion - Impaired PINK1/Parkin-mediated mitophagy	- Peroxynitrite formation→NO inactivation - mtDNA acting as DAMPs → TLR9 and cGAS-STING activation - NF-κB signaling and inflammasome activation - Accumulation of fragmented mitochondria and apoptotic signaling	- Endothelial dysfunction: ↓ NO bioavailability, ↑ permeability - Pro-inflammatory, pro-thrombotic phenotype - Leukocyte adhesion and vascular activation, initiating atherogenesis	[8,20,21,22,23,24,25,26,27,28,29,30]
Vascular Smooth Muscle Cells (VSMCs)	- Metabolic reprogramming: OXPHOS → aerobic glycolysis (Warburg-like effect) - ↑ Mitochondrial mROS during glycolysis shift - Dynamics imbalance: excessive fission→apoptosis and senescence	- mROS-driven activation of MAPK and NF-κB pathways - Enhanced protein and ECM synthesis - Fission-mediated loss of mitochondrial integrity	- Phenotypic switch: contractile → synthetic - Intimal migration and fibrous cap formation - Excess apoptosis → fibrous cap thinning, plaque instability and rupture	[31,32,33,34,35,36,37]
Macrophages	- M1 phenotype: glycolytic metabolism, impaired respiration, ↑ mROS - M2 phenotype suppressed due to OXPHOS impairment - mtDNA release amplifying inflammatory signaling - Defective mitophagy (impaired PINK1-Parkin axis)	- NF-κB activation, NLRP3 inflammasome assembly → IL-1β release - Type I interferon responses triggered by mtDNA - Accumulation of dysfunctional mitochondria and lipid droplets	- Pro-inflammatory macrophage dominance - Foam cell transformation - Necrotic core expansion and chronic inflammation, destabilizing plaques	[38,39,40,41]
Shared Pathways Across Cell Types	- mROS overproduction - mtDNA damage and release - Impaired mitochondrial dynamics - Defective mitophagy	- Activation of redox-sensitive inflammatory pathways (NF-κB, MAPK) - Inflammasome activation - Metabolic rewiring - Apoptosis/senescence	- Global amplification of vascular inflammation - Impaired tissue homeostasis - Structural destabilization of atherosclerotic lesions	[21,22,24,26,27,28,29,34,35,39,40]

cGAS-STING = cyclic GMP–AMP synthase–stimulator of interferon genes; DAMPs = damage-associated molecular patterns; Drp1 = dynamin-related protein 1; ECM = extracellular matrix; ETC = electron transport chain; IL-1β = interleukin-1β; MAPK = mitogen-activated protein kinase; MFN1 = mitofusin-1; MFN2 = mitofusin-2; mROS = mitochondrial reactive oxygen species; mtDNA = mitochondrial DNA; NLRP3 = pyrin domain-containing protein 3; NF-κB = nuclear factor κB; PINK1 = PTEN-induced putative kinase 1; Parkin = Parkin RBR E3 ubiquitin ligase; TLR9 = Toll-like receptor 9; ↑ = increase; ↓ = decrease; → = leads-to.

### 2.2. Mitochondrial Reprogramming of VSMCs: From Contractile Guardians to Drivers of Plaque Instability

VSMCs, the principal constituents of the medial layer of the arterial wall, are remarkably plastic. During atherogenesis, they undergo a profound phenotypic switch from a differentiated, contractile state to a proliferative, synthetic phenotype [31]. This transition is closely linked to mitochondrial function, which dictates cellular energy metabolism, redox balance, and susceptibility to apoptosis.

In the atherosclerotic milieu, VSMCs frequently undergo metabolic reprogramming, shifting from OXPHOS toward aerobic glycolysis [32]. This shift—reminiscent of the Warburg effect in cancer cells—sustains the anabolic demands of synthetic VSMCs, including protein synthesis, extracellular matrix (ECM) deposition, and migration into the intima [33]. While initially adaptive, this metabolic remodeling fosters lesion expansion and fibrous cap formation at the cost of pathological consequences. Elevated glycolytic flux is commonly accompanied by excess mROS generation, which activates redox-sensitive pathways such as mitogen-activated protein kinase (MAPK) and NF-κB, thereby amplifying VSMC proliferation and inflammation [34,35].

Disruption of mitochondrial dynamics further aggravates disease progression. A shift favoring fission over fusion renders VSMCs prone to apoptosis, weakening the fibrous cap and predisposing plaques to rupture [36]. The accumulation of apoptotic and senescent VSMCs undermines plaque stability, positioning mitochondrial dysregulation as a central mechanism linking lesion growth with destabilization [37].

### 2.3. Mitochondrial Determinants of Macrophage Function: Inflammatory Signaling and Foam Cell Transformation

**Figure 1:**
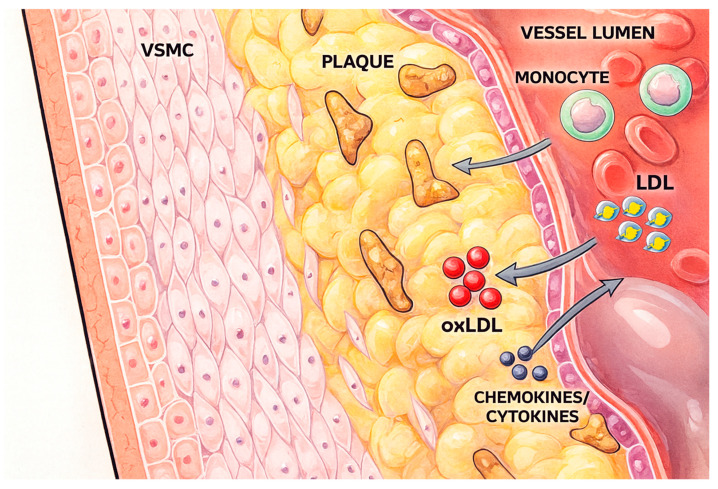
Atherogenesis and foam cell transformation. atherogenesis and foam cell transformation-low-density lipoproteins (LDLs) penetrate the endothelial layer and undergo oxidative modification within the vessel wall. Circulating monocytes are recruited by endothelial-derived chemokines and differentiate into macrophages, which internalize oxidized LDLs (oxLDLs) and transform into lipid-laden foam cells. The accumulation of foam cells, together with VSMCs and inflammatory mediators, drives the development of atherosclerotic plaques, a hallmark of vascular disease progression.

Macrophages are pivotal in atherogenesis, orchestrating both inflammatory amplification and resolution within the plaque microenvironment (Figure 1).

Their phenotypic polarization is tightly coupled to mitochondrial metabolism. Classically activated M1 macrophages adopt a glycolytic profile and display impaired mitochondrial respiration, marked by ETC disruption and elevated mROS generation [38]. Excess mROS activates NF-κB signaling and promotes assembly of the NOD-, LRR-, and pyrin domain-containing protein 3 (NLRP3) inflammasome, leading to caspase-1 activation and maturation of interleukin-1β (IL-1β), a cytokine that drives vascular inflammation and destabilizes plaques.

In contrast, alternatively activated M2 macrophages rely on intact OXPHOS and fatty acid oxidation to sustain anti-inflammatory and reparative functions [39]. Within the atherosclerotic plaque, however, mitochondrial dysfunction frequently impairs M2 polarization, skewing macrophage populations toward a pro-inflammatory state. Release of mtDNA from damaged organelles further amplifies inflammation by acting as a potent DAMP, activating cytosolic DNA sensors and type I interferon responses [40].

Defective mitophagy—often due to impaired recruitment of autophagy machinery or disruption of the PINK1–Parkin pathway—exacerbates this process, allowing dysfunctional mitochondria to accumulate. This not only fuels persistent cytokine production but also promotes lipid uptake and foam cell transformation, consolidating macrophage-driven inflammation and contributing to necrotic core formation. Taken together, mitochondrial integrity emerges as a critical determinant of macrophage phenotype, dictating whether these cells stabilize or destabilize the atherosclerotic plaque [41].

## 3. Molecular Pathways Involved in Mitochondrial Reprogramming

Mitochondrial reprogramming in AS relies on the coordinated regulation of a limited set of fundamental processes that determine mitochondrial quality and adaptability. These include biogenesis, which expands the mitochondrial network and replenishes functional organelles; dynamics, the balance of fusion and fission that preserves structural and functional integrity; mitophagy, the selective removal of damaged mitochondria; and metabolic signaling, which integrates mitochondrial activity with cellular and vascular homeostasis [10]. Together, these pathways form an interconnected framework that defines how mitochondria adapt—or fail to adapt—to atherogenic stressors.

### 3.1. The SIRT1–PGC-1α Pathway in VSMC Mitochondrial Function and Vascular Remodeling

Sirtuin 1 (SIRT1) and PGC-1α are central regulators of energy metabolism, traditionally attributed to their control of nuclear transcriptional programs. An emerging and less well-characterized dimension of SIRT1 biology concerns its extranuclear localization, particularly within mitochondria [42]. Pioneering work by Aquilano and colleagues, employing confocal microscopy and subcellular fractionation, first demonstrated that both SIRT1 and the transcriptional coactivator PGC-1α localize to mitochondria in human cell lines and platelets, as well as across multiple murine tissues [43]. Acting synergistically, SIRT1 and PGC-1α promote mitochondrial biogenesis and enhance bioenergetic efficiency. Mechanistically, SIRT1-mediated deacetylation of PGC-1α potentiates its transcriptional coactivator activity, thereby driving the expression of nuclear-encoded genes involved in OXPHOS, antioxidant defense, and the coordination of mitochondrial replication and turnover [44].

Both SIRT1 and PGC-1α are expressed in VSMCs, implicating them as central modulators of vascular bioenergetics and redox balance [45]. Experimental evidence demonstrates that SIRT1 overexpression in VSMCs mitigates angiotensin II (AngII)-induced hypertension, vascular remodeling, and associated pathological alterations in murine models [46]. Furthermore, elevated SIRT1 levels suppress neointimal hyperplasia by inhibiting VSMC proliferation and migration [47]. This inhibitory effect has been linked to the pro-apoptotic signaling molecule Fas ligand (FasL), suggesting that modulation of apoptotic pathways contributes to SIRT1-mediated attenuation of neointima formation [48].

In parallel, reduced neovascularization of the vessel wall has been associated with suppression of neointimal formation in early hypercholesterolemia-driven atherosclerosis [49]. Mechanistic studies indicate that SIRT1 represses hypoxia-induced angiogenesis through deacetylation and inactivation of hypoxia-inducible factor-1α (HIF-1α), even under low oxygen conditions [50]. Consistently, SIRT1-dependent inhibition of HIF-1α expression in hypoxic VSMCs has been shown to contribute to the repression of neointimal formation [51].

### 3.2. Mitochondrial Fusion and Fission Dynamics in Vascular Cells

Mitochondrial fission is essential for fundamental cellular processes, including cell division. Symmetrical (replicative) fission produces two functional daughter mitochondria and is tightly coupled with the cell cycle, whereas asymmetrical fission facilitates the segregation of damaged components destined for mitophagy [52]. The dynamin-related protein 1 Drp1 is the central mediator of mitochondrial fission. While predominantly cytosolic, a substantial fraction of Drp1 localizes to mitochondria, where its activity depends on four structural domains: the N-terminal GTPase domain, the middle helical domain, the variable insert B domain, and the C-terminal GTPase effector domain (GED). Deletion of the GTPase, middle, or GED markedly impairs Drp1 function [53]. Mutations disrupting GTP binding prevent proper division, resulting in elongated and interconnected mitochondria [54]. The middle domain appears critical for Drp1 oligomerization and the formation of spiral ring assemblies on membranes, and mutations within this region significantly alter its activity [55].

Drp1-driven fission is further modulated by outer membrane adaptors such as fission protein 1 (FIS1) and mitochondrial fission factor (MFF), which recruit Drp1 to sites of mitochondrial division and enable the selective removal of dysfunctional segments [56]. Excessive fission has been implicated in endothelial dysfunction, VSMC phenotypic switching, and plaque instability [41].

Conversely, mitochondrial fusion proceeds as a sequential two-step process involving independent merging of the outer and inner membranes. This is mediated by MFN1 and MFN2 on the outer mitochondrial membrane, and by optic atrophy protein 1 (OPA1) on the inner membrane and intermembrane space [57]. Mitofusins, anchored by transmembrane and C-terminal sequences, form antiparallel homo- or heterodimeric coiled-coil interactions that tether adjacent mitochondria and initiate fusion [58]. Beyond mitochondria, MFN2 localizes to the endoplasmic reticulum (ER), where it regulates ER morphology, promotes ER–mitochondria tethering, and enhances mitochondrial calcium uptake [59]. OPA1, existing in at least eight splice variants with distinct fusion efficacy and proteolytic sensitivity, is critical for inner membrane fusion and cristae maintenance [60].

Fusion–fission balance is also modulated by bioactive lipids. Phosphatidic acid, generated by mitochondrial phospholipase D, promotes fusion by inducing membrane curvature and recruiting adaptor proteins [61], whereas its hydrolysis by lipin-1 generates diacylglycerol, a lipid species that promotes fission [62].

Pharmacological inhibition of Drp1 and fusion-promoting strategies are currently under investigation as approaches to restore mitochondrial morphology, reduce apoptotic signaling, and preserve vascular cell function [63].

### 3.3. Selective Mitochondrial Clearance: Mitophagy in Atherosclerosis and Vascular Homeostasis

Mitophagy, the selective autophagic elimination of mitochondria, is a fundamental process for maintaining mitochondrial fitness across cell types and represents a cornerstone of organelle quality control [64]. This pathway operates through ubiquitin-dependent and -independent mechanisms.

The ubiquitin-dependent route is primarily governed by the PTEN-induced kinase 1 (PINK1)–Parkin axis [65]. Under basal conditions, PINK1 is constitutively imported into mitochondria via the TOM/TIM complexes, where it is cleaved by Presenilin-associated rhomboid-like (PARL) protease and rapidly degraded [66]. Mitochondrial depolarization halts this import, leading to PINK1 accumulation on the outer membrane [67]. There, PINK1 autophosphorylates and phosphorylates both Parkin and ubiquitin, thereby driving Parkin recruitment and full activation [68]. Activated Parkin ubiquitinates numerous outer membrane proteins, which are recognized by LC3-interacting ubiquitin-binding receptors such as p62 and NBR1. These adaptor proteins mediate sequestration of damaged mitochondria into autophagosomes, which subsequently fuse with lysosomes for degradation and recycling [69,70].

By contrast, ubiquitin-independent mitophagy relies on LC3-interacting region (LIR)-containing proteins such as BNIP3, NIX (BNIP3L), and FUNDC1 [71]. These receptors tether damaged mitochondria directly to LC3-positive autophagosomes, bypassing ubiquitin signaling. Their activity is particularly prominent under hypoxia or nutrient deprivation, often regulated by transcription factors including HIF-1α [72]. For example, hypoxia-induced BNIP3 and NIX oligomerization enhances interaction with LC3, thereby promoting mitophagic flux [73]. Notably, BNIP3 and NIX also exhibit crosstalk with the PINK1–Parkin pathway, revealing an integrated network that ensures mitochondrial turnover under diverse stress conditions [74].

In the context of atherosclerosis, defective mitophagy has profound vascular consequences. The persistence of dysfunctional mitochondria within endothelial cells leads to excessive ROS release, mitochondrial DNA leakage, and activation of NLRP3-dependent inflammation, thereby compromising endothelial integrity [75]. Consistent with the concept that successful mitophagy protects against oxidative stress and from the release of proteins that participate in cell death pathways, Swiader et al. showed that mitophagy safeguarded human VSMC against oxidized LDL-induced apoptosis [76]. In macrophages, defective PINK1–Parkin–mediated mitophagy promotes lipid accumulation and foam cell formation, accelerating necrotic core expansion [77]. Collectively, these mechanisms link impaired mitochondrial turnover directly to vascular inflammation, remodeling, and plaque vulnerability.

## 4. Therapeutic Strategies Targeting Mitochondrial Dysfunction in Atherosclerosis

Recognition of mitochondrial dysfunction as a central driver of AS has catalyzed the development of therapeutic approaches aimed at preserving mitochondrial integrity, limiting oxidative stress, and protecting the vascular endothelium from atherogenic injury. Current strategies span small-molecule antioxidants, pharmacological agents, gene-based interventions, and advanced nanotechnologies [78].

### 4.1. Antioxidants and Pharmacological Compounds

Mitochondria-targeted antioxidants have demonstrated potent effects in modulating oxidative stress, thereby influencing key mechanisms of CVD pathogenesis [79]. Among these, MitoQ is the most extensively studied. Its quinone antioxidant moiety, conjugated to a lipophilic triphenylphosphonium cation, enables selective mitochondrial accumulation [80]. Preclinical studies show that four weeks of oral MitoQ supplementation fully restore endothelium-dependent dilation in aged mice to levels observed in young controls [81]. Translating these findings, clinical trials in middle-aged and older adults demonstrated improved endothelial function, reduced aortic stiffness in subjects with elevated baseline values, and decreased systemic oxidative stress, without changes in blood pressure, inflammation, or traditional risk factors [82]. Beyond vascular effects, MitoQ has been shown to preserve mitochondrial integrity in tubular cells under hyperglycemia by enhancing mitophagy via the nuclear factor erythroid 2-related factor 2 (Nrf2)/PINK1 pathway, which Nrf2 directly controls PINK1 transcription under oxidative stress leading to PINK1 accumulation on damaged mitochondria, recruitment of Parkin, ubiquitination of outer membrane proteins, and selective autophagic clearance of dysfunctional mitochondria [83].

Resveratrol, another well-characterized compound, attenuates endothelial dysfunction and atherogenesis by mitigating hydrogen peroxide- and catechol estrogen-induced cytotoxicity and limiting mROS accumulation [84]. At concentrations of 25–50 μM, it confers robust cytoprotection without detectable toxicity [85]. Mechanistically, resveratrol: (i) competes with coenzyme Q at mitochondrial complex III to attenuate electron leakage [86]; (ii) boosts glutathione levels, preserving cell viability [87]; and (iii) upregulates endogenous antioxidant and detoxifying enzymes in cardiomyocytes [88].

Metformin, widely used in type 2 diabetes mellitus, exerts cardiovascular benefits beyond glycemic control [89]. It slows progression of carotid intima–media thickness and reduces myocardial infarction incidence in diabetic patients [90]. Mechanistically, metformin inhibits mitochondrial fragmentation via AMP-activated protein kinase (AMPK) activation, suppressing endothelial apoptosis and inflammation [91]. Similarly, fish oil, another AMPK activator, modulates expression of mitochondrial dynamics–related proteins (MFN2, Fis1) and reduces plaque burden in ApoE^−^/^−^ mice fed a high-fat diet [92].

Triphenylphosphonium chloride (Mito-TEMPO) is a physicochemical compound belonging to the class of superoxide dismutase (SOD) mimetics. It readily traverses lipid bilayers and selectively accumulates within mitochondria. Evidence from both in vitro and in vivo studies demonstrates that Mito-TEMPO functions as a mitochondria-targeted antioxidant with potent superoxide and alkyl radical–scavenging activity [93,94]. An in vitro study demonstrated that mito-TEMPO incubation prevented pharmacologically induced manganese superoxide dismutase (MnSOD)-inhibition–triggered cell death in adult cardiomyocyte [95]. In vivo studies demonstrated that mito-TEMPO administration enhanced cardiac function in a murine model of pressure-overload–induced heart failure [96] and mitigated diabetes-associated cardiac injury and post-infarction mortality [97]. Beyond its canonical role in ATP synthesis, Coenzyme Q_10_ (CoQ10), as a lipid-soluble quinone residing in the inner mitochondrial membrane, exerts multifaceted protective effects in atherosclerosis through both bioenergetic and redox-signalling pathways. Supplementation with CoQ_10_ has been shown to activate AMPK and upregulates OPA1 preserving mitochondrial membrane potential and ATP synthesis. By boosting ATP production, CoQ_10_ helps restore energy homeostasis in vascular endothelial cells exposed to pro-atherogenic stimuli (e.g., oxLDL) [98]. Concurrently, CoQ_10_ supports lipid metabolism regulation: by modulating the mevalonate pathway, it helps restore balance in cholesterol biosynthesis, upregulates LDL receptors, downregulates proprotein convertase subtilisin/kexin type 9 (PCSK9), and normalizes sterol regulatory element-binding proteins 2 (SREBP-2) feedback via INSIG1 and INSIG2, thereby reducing intracellular cholesterol overload [99]. Additionally, CoQ_10_ contributes to redox homeostasis by increasing endogenous antioxidants (e.g., glutathione, SOD) and by inhibiting inflammasome activation triggered by mitochondrial dysfunction [100]. Through this integrated network of mitochondrial bioenergetics, redox signaling, lipid regulation, and inflammation control, the antioxidants and pharmacological compounds emerge not merely as a classical antioxidants but as a mitochondria-targeted therapeutic agent with translational potential to slow or reverse the progression of atherosclerotic disease.

### 4.2. Gene-Based Interventions

Cutting-edge advances in mitochondrial gene-targeted interventions are opening new therapeutic frontiers in AS. CRISPR/Cas9-based approaches have been used to eliminate specific mtDNA mutations, such as the m.15059G>A variant in *MT-CYB* gene, thereby correcting defective mitophagy and lipid metabolism in macrophages, with consequent reduction in plaque formation [101].

At the post-transcriptional level, circHIPK3, markedly upregulated in vulnerable plaques, promotes VSMC necroptosis via direct targeting of DRP1, enhancing mitochondrial fission, mROS generation, and necroptotic death. Silencing circHIPK3 in ApoE^−^/^−^ mice significantly attenuates plaque formation and progression [102]. Similarly, C1q/TNF-related protein 9 (CTRP9) protects macrophages from ox-LDL–induced dysfunction by enhancing autophagic flux and reducing lipid accumulation. This effect is mediated through ubiquitin specific peptidase 22 (USP22)-dependent stabilization of SIRT1, establishing a novel CTRP9–USP22–SIRT1 axis relevant to AS [103].

Systems-level approaches are also providing new insights. In a cross-disease transcriptomic study, Wang et al. identified 13 mitochondria-associated genes dysregulated in both systemic sclerosis and AS. Refinement by machine learning highlighted *IFN-α inducible gene 6* (*IFI6*), *Fascin actin-bundling protein 1* (*FSCN1*) and *α sarcoglycan* (*SGCA*) as hub genes with strong diagnostic potential (AUC ≈ 0.90) across independent datasets [104]. These findings propose a molecular diagnostic framework that extends beyond lipid-centric paradigms.

### 4.3. Nanotechnologies for Mitochondrial-Targeted Drug Delivery

Nanoparticle-based drug delivery systems represent a rapidly expanding area in mitochondrial therapeutics. Polymeric nanoparticles, liposomes, and inorganic nanostructures can be engineered for precise size, charge, and surface functionalization (e.g., PEGylation), optimizing biodistribution, controlled release, and immune evasion [105]. Despite these advances, challenges remain, including achieving tissue specificity, preventing opsonization, and improving drug solubility and bioavailability [106].

Several mitochondria-targeted peptides exploit the organelle’s negative membrane potential. Szeto–Schiller (SS) peptides and mitochondria-penetrating peptides (MPPs) accumulate >1000-fold within the inner membrane by alternating cationic and aromatic residues [107,108]. Among them, elamipretide (SS-31) is the most advanced candidate, shown to modulate membrane potential, improve OXPHOS, reduce mROS, and confer cardioprotection in models of heart failure, ischemia–reperfusion injury, and arrhythmias [109].

Similarly, triphenylphosphonium (TPP) is a small, lipophilic cation with ~1000-fold mitochondrial accumulation, widely used for conjugation to antioxidants such as MitoQ [110,111]. Another strategy involves mitochondrial targeting signal peptides (MTSs), 20–40 amino acids long, which direct cargo into mitochondria but require conjugation to cell-penetrating peptides (CPPs) for efficient uptake and endosomal escape [112,113].

Beyond carriers, mitochondria-derived peptides—including Humanin, mitochondrial ORF of the 12S rRNA Type-C (MOTS-c), and small humanin-like peptides (SHLPs)—are themselves bioactive, exerting antioxidant, anti-apoptotic, and anti-inflammatory effects linked to CVD risk modulation [114].

Finally, polymeric nanoparticles such as poly(lactic-co-glycolic acid) (PLGA) formulations have been applied to deliver mitochondrial modulators in cardiovascular models. Delivery of mitochondrial division inhibitor 1 (Mdivi1) via PLGA nanoparticles reduced infarct size by >30% in ischemia–reperfusion injury, while quercetin-loaded PLGA nanoparticles decreased mROS and improved calcium buffering in hypoxia–reoxygenation cardiomyocytes [115,116]. Dual-pathway strategies further enhanced efficacy: combined delivery of cyclosporin A (CsA) and pitavastatin-loaded nanoparticles reduced infarct size by an additional 10–15% compared to single agents, while limiting overall cell death [117].

A comparative synthesis of these mitochondria-targeted therapeutic modalities—encompassing small molecules, peptides, and nanotechnology-based carriers—is provided in Table 2, which highlights their mechanisms of action, preclinical and clinical evidence, and translational potential in AS.

### 4.4. Mitochondria-Targeted Redox Therapeutics as Emerging Stabilizers of the Atherosclerotic Plaque

mROS drive a distinct layer of macromolecular injury in atherosclerosis by oxidizing mitochondrial DNA, disrupting respiratory-chain proteins, and inducing lipid peroxidation that amplifies redox-driven signaling loops [118]. In endothelial cells, this damage compromises nitric-oxide bioavailability, promotes maladaptive unfolded-protein responses, and accelerates endothelial-to-mesenchymal transition, collectively lowering barrier integrity and favoring leukocyte recruitment [119]. In vascular smooth muscle cells, mROS-induced genomic and mitochondrial instability pushes a shift from a contractile to a synthetic, pro-inflammatory phenotype with impaired mitochondrial dynamics that reinforce proliferative and migratory behavior [120]. Within atherosclerotic plaques, these converging phenotypic alterations undermine fibrous-cap architecture by reducing collagen synthesis, increasing matrix-degrading enzyme expression, and fostering necrotic-core expansion [121]. The resulting microenvironment couples metabolic fragility to structural vulnerability, positioning mROS as a central—yet targetable—driver of plaque destabilization in advanced disease. Emerging therapeutic strategies aimed at interrupting mROS-driven macromolecular injury in atherosclerosis now converge on precise mitochondrial stabilization, nucleic-acid protection, targeted suppression of lipid peroxidation, and restoration of proteostasis. Cardiolipin-stabilizing peptides such as elamipretide (SS-31, MTP-131, Bendavia) restructure inner-membrane architecture to preserve electron-transport efficiency, limit ROS amplification and improve mitochondrial bioenergetics in disease models [122], offering a tractable route to prevent downstream endothelial and smooth-muscle dysfunction. Parallel approaches that protect mtDNA—including mitochondrial transcription factor A (TFAM)-based strategies and engineered mitochondria that enhance mtDNA packaging, repair or selective replacement—aim to blunt mtDNA release and cGAS/STING-driven inflammation while preserving organellar translation and respiratory competence [123,124]. Pharmacological blockade of lipid peroxidation with radical-trapping antioxidants such as prevents polyunsaturated fatty acid (PUFA)-phospholipid oxidation, limits ferroptotic and non-apoptotic lipid-driven cell death in vascular cells and has shown efficacy in preclinical atherosclerosis models [125,126]. Finally, interventions directed at protein carbonylation and impaired proteostasis—ranging from small molecules that reduce irreversible carbonyl adduct formation to enhancers of selective autophagy and proteasomal clearance—seek to restore functional proteomes within endothelium and smooth muscle, thereby reducing maladaptive phenotype switching and matrix degradation [127]. Together, these complementary modalities transform conceptual antioxidant therapy into mechanism-specific, mitochondria-centric regimens that can be rationally combined with lipid-lowering and anti-inflammatory treatments to stabilise plaques and limit progression to rupture.

**Table 2:** Comparative overview of mitochondria-targeted therapeutic strategies in atherosclerosis.

Category	Agent/Approach	Molecular Mechanism	Evidence	Therapeutic Outcome	References
Antioxidants/Pharmacological Compounds	MitoQ	- Selective mitochondrial accumulation via TPP^+^ - Activation of Nrf2/PINK1 pathway - Promotion of mitophagy; scavenging of mROS; preservation of ETC integrity	- Preclinical: aged mice, restoration of endothelium-dependent dilation - Clinical: ↑ endothelial function, ↓ aortic stiffness, ↓ systemic oxidative stress	Endothelial protection, preservation of mitochondrial integrity, attenuation of oxidative stress	[80,81,82,83]
	Resveratrol	- Competitive inhibition at mitochondrial complex III - ↑ glutathione - upregulation of phase II detoxifying/antioxidant enzymes - mitigation of H_2_O_2_- and catechol estrogen-induced cytotoxicity	In vitro (25–50 μM): ↓ intra-/extracellular mROS, no cytotoxicity	Cytoprotection, improved redox balance, attenuation of oxidative stress	[84,85,86,87,88]
	Metformin	AMPK activation; inhibition of mitochondrial fragmentation; anti-apoptotic and anti-inflammatory; modulation of mitochondrial dynamics	- Preclinical: endothelial protection - Clinical: ↓ carotid intima–media thickness, ↓ myocardial infarction incidence	Cardiovascular protection in T2DM, anti-atherogenic effects	[89,90,91]
	Fish oil	AMPK activation; ↑ MFN2, ↓ Fis1; modulation of mitochondrial dynamics	ApoE^−^/^−^ mice on high-fat diet: ↓ plaque burden	Improved mitochondrial dynamics, reduced lesion progression	[92]
	Mito-TEMPO	SOD mimetic; scavenging of superoxide and alkyl radicals; selective mitochondrial accumulation	- In vitro: prevents MnSOD-inhibition–induced cell death - In vivo: enhanced cardiac function, reduced diabetes-associated injury	Reduction in mROS, protection against mitochondrial oxidative damage	[93,94,95,96,97]
	CoQ10	activation of AMPK; upregulation of OPA1, preserving mitochondrial membrane potential and ATP synthesis; restoration of energy homeostasis in endothelial cells; modulation of mevalonate pathway, ↑ LDL receptors, ↓ PCSK9, normalization of SREBP-2 feedback; ↑ endogenous antioxidants (glutathione, SOD); inhibition of inflammasome activation	- Preclinical: endothelial cells exposed to oxLDL - Clinical supplementation studies show improved mitochondrial function and reduced oxidative stress	Restoration of endothelial bioenergetics, improved lipid metabolism, redox homeostasis, attenuation of inflammasome-driven inflammation	[98,99,100]
Gene-based Interventions	CRISPR/Cas9	Removal/correction of mtDNA mutations (e.g., MT-CYB m.15059G>A); restoration of mitophagy and lipid metabolism	Preclinical: macrophages/monocytes	Attenuation of plaque formation, improved mitochondrial function	[101]
	circHIPK3 silencing	Inhibition of DRP1-mediated fission; ↓ mitochondrial fragmentation; ↓ mROS; prevention of VSMC necroptosis	ApoE^−^/^−^ mice: ↓ plaque progression	Vascular protection, preservation of fibrous cap integrity	[102]
	CTRP9–USP22–SIRT1 axis	USP22-mediated stabilization of SIRT1; ↑ autophagic flux; ↓ lipid accumulation in macrophages	In vitro: human macrophages + oxLDL	Preservation of macrophage reparative function under atherogenic stress	[103]
Nanotechnologies/Mitochondria-targeted Delivery	Mitochondrial biomarkers (IFI6, FSCN1, SGCA)	Shared DEGs in AS and systemic sclerosis; diagnostic potential	Multi-cohort transcriptomics, AUC ≈ 0.90	Highly sensitive and specific molecular diagnosis for early detection of mitochondrial dysfunction in AS	[104]
	SS-peptides (Elamipretide/SS-31)	>1000× mitochondrial accumulation; cardiolipin stabilization; ↑ OXPHOS; ↓ mROS; improved Ca^2+^ handling; protection of mtDNA	Preclinical and clinical: heart failure, ischemia–reperfusion models	Restoration of mitochondrial bioenergetics, cardioprotection, plaque stabilization	[107,108,109]
	TPP-conjugates (e.g., MitoQ)	Lipophilic cation-driven mitochondrial targeting; conjugation of antioxidants; reduces lipid peroxidation	Widely tested with antioxidant cargo	Targeted mitochondrial delivery, reduction in mROS-mediated injury	[110,111]
	MTS + CPP constructs	Dual mitochondrial targeting; enhanced cell permeability; efficient mitochondrial delivery	In vitro: ↑ cellular uptake, efficient mitochondrial import	Enhanced delivery of therapeutic proteins/peptides, improved mitochondrial repair	[112,113]
	Mitochondrial-derived peptides (MOTS-c, SHLPs, Humanin)	Antioxidant, anti-apoptotic, anti-inflammatory; restoration of proteostasis	Association studies with cardiovascular outcomes	Cardioprotection, modulation of CVD risk factors	[114]
	PLGA nanoparticles (Mdivi1, quercetin, CsA, pitavastatin)	Controlled drug release; inhibition of mitochondrial fission (Mdivi1); ↓ mROS; protection of mitochondrial membrane integrity; modulation of mitochondrial permeability transition	Preclinical I/R models: ↓ infarct size > 30%; dual-drug delivery: additional ↓ 10–15% cell death	Reduction in oxidative stress, improved mitochondrial function, anti-inflammatory effects	[115,116,117]
Emerging Mitochondria-Targeted Redox Therapies	Cardiolipin-stabilizing peptides	Preservation of inner membrane architecture; improved ETC efficiency; ↓ mROS amplification	Preclinical: models of ischemia–reperfusion, heart failure	Restoration of mitochondrial bioenergetics, protection against oxidative stress	[122]
	mtDNA protection strategies (TFAM-based, engineered mtDNA)	Prevention of mtDNA release; maintenance of mitochondrial transcription/translation; suppression of cGAS/STING-mediated inflammation	Preclinical models of atherosclerosis	Reduction in vascular inflammation, preservation of mitochondrial function	[123,124]
	Lipid peroxidation blockers (radical-trapping antioxidants)	Inhibition of PUFA-phospholipid oxidation; prevention of ferroptosis and lipid-driven cell death	Preclinical: atherosclerosis models	Protection of vascular cells, reduced necrotic core formation	[125,126]
	Proteostasis-targeting interventions	Reduction in protein carbonylation; enhancement of selective autophagy and proteasomal clearance	Preclinical: endothelial and VSMC models	Restoration of functional proteomes, attenuation of maladaptive phenotype switching	[127]

AMPK = AMP-activated protein kinase; CCP = cell-penetrating peptides; CoQ10 = Coenzyme Q_10_; CsA = cyclosporin A; CTRP9 = C1q/TNF-related protein 9; CVD = cardiovascular disease; DRP1 = dynamin-related protein 1; ETC = electron transport chain; Fis1 = fission protein 1; FSCN1 = Fascin actin-bundling protein 1; IFI6 = IFN-α inducible gene 6; LDL = low-density lipoprotein; MFN2 = mitofusin-2; Mdivi1 = mitochondrial division inhibitor 1; MitoQ = Mitoquinone; Mito-TEMPO = Triphenylphosphonium chloride; MnSOD = Manganese Superoxide Dismutase; MOTS-c = mitochondrial ORF of the 12S rRNA Type-C; mROS = mitochondrial ROS; MTS = mitochondrial targeting signal peptides; OPA1 = optic atrophy protein 1; oxLDL = oxidized LDL; OXPHOS = oxidative phosphorylation; PCSK9 = proprotein convertase subtilisin/kexin type 9; PLGA = poly(lactic-co-glycolic acid); PUFA= polyunsaturated fatty acid; SOD = superoxide dismutase; SGCA = α sarcoglycan; SHLPs = small humanin-like peptides; SIRT1 = sirtuin 1; SREBP-2 = sterol regulatory element-binding proteins 2; SS-peptides = Szeto–Schiller peptides; TFAM = mitochondrial transcription factor A; TTP = triphenylphosphonium; USP22 = ubiquitin specific peptidase 22; VSMC = vascular smooth muscle cells; mtDNA = mitochondrial DNA; ↑ = increase; ↓ = decrease.

## 5. Clinical Implications and Future Directions

Recognition of mitochondrial dysfunction as a central element in AS pathogenesis not only deepens our mechanistic understanding of vascular biology but also opens promising avenues for clinical translation. The concept of reprogramming mitochondrial function to achieve vascular protection heralds a paradigm shift—from traditional therapies centered on lipid lowering and systemic inflammation to a more organelle-focused strategy targeting the root of cellular dysfunction. Despite this promise, the clinical assessment of mitochondrial health remains a substantial challenge [128].

### 5.1. Precision Therapeutics: High-Resolution Molecular Imaging, Omics Based Approach

Emerging technologies in precision medicine are redefining the therapeutic landscape of mitochondrial dysfunction. Positron emission tomography (PET) remains the most sensitive modality for detecting molecular signatures of oxidative stress. The development of innovative tracers, such as [^18^F]ROStrace, which selectively localizes to mROS-producing mitochondria, allows for early detection of oxidative stress in tissues, even at subclinical stages, before irreversible damage occurs [129]. Complementing these approaches, Wu et al. introduced a novel mitochondria-targeted near-infrared probe (AS-CO), capable of detecting carbon monoxide (CO) fluctuations independently of metal ion mediation. AS-CO enables dynamic visualization of CO levels in living cells and in murine models of AS, demonstrating clinical potential for monitoring disease onset, progression, and therapeutic response [130].

Genomics has also emerged as a powerful tool for risk stratification, particularly in families with a strong history of AS. Mutations in genes such as ApoA5, CETP, and LPL substantially increase susceptibility by altering cholesterol metabolism and absorption [131,132,133]. Transcriptomic profiling provides complementary insights, systematically identifying dysregulated genes and signaling pathways implicated in endothelial dysfunction, inflammation, and oxidative stress [134]. Integrated transcriptomic–metabolomic studies reveal that disruption of glycolytic metabolism in vascular ECs leads to impaired angiogenesis, reduced cGMP, and adoption of a pro-inflammatory phenotype [135]. Shear stress adaptation is another area of focus: transcriptomic studies have identified 86 shear-sensitive genes defining an intermediate endothelial phenotype that may represent early events in AS pathogenesis [136].

At the epitranscriptomic level, N^6^-methyladenosine (m^6^A) modifications have been implicated in AS progression. Oscillatory shear stress downregulates the methyltransferase METTL3, destabilizing EGFR mRNA and thereby exacerbating endothelial dysfunction. Conversely, METTL3-mediated m^6^A stabilization of EGFR mRNA attenuates lesion development, underscoring the therapeutic potential of RNA modifications [137].

Proteomic strategies further expand this precision framework. In ECs, ox-LDL binding to LOX-1 promotes lipid accumulation, inflammatory activation, and ECM remodeling. Mass spectrometry identified Rho pathway components (ARHGEF1, ROCK2) as LOX-1 interactors, with ROCK2 activation shown to drive NF-κB signaling and IL-8 release—effects reversed by pharmacological inhibition [138]. Similarly, Goettsch et al. demonstrated that OSCAR, a receptor classically linked to bone homeostasis, promotes oxidative stress–mediated AS by activating STAT signaling cascades and altering EC adhesion [139]. Collectively, these high-resolution molecular technologies integrate imaging, genomics, transcriptomics, epitranscriptomics, and proteomics, offering a multidimensional framework for precision therapeutics in AS.

Sex-specific considerations also deserve attention within this precision framework. Although lipid-lowering therapy did not differ substantially between sexes, Gavina et al. found that women displayed higher total and LDL cholesterol levels, together with higher HDL concentrations, yet a greater prevalence of AS [140]. These findings likely reflect a complex interplay between age-related metabolic remodeling, reduced HDL functionality, and residual inflammatory risk that attenuates the protective effects of HDL in elderly women [141]. Recent evidence indicates that qualitative HDL dysfunction and differences in triglyceride-rich lipoproteins may underlie the higher atherosclerotic burden in women despite comparable treatment exposure [142].

### 5.2. Lifestyle-Based Mitochondrial Reprogramming: Foundational and Synergistic Strategies

While pharmacological strategies hold promise, lifestyle and behavioral interventions remain the cornerstone of vascular protection [143,144]. Physical activity, in particular, exerts powerful mitohormetic effects—stimulating mitochondrial biogenesis, enhancing antioxidant defense, and improving dynamics [145]. Regular exercise reduces cardiovascular morbidity and mortality by up to 44% [146], extends lifespan [147], and promotes healthy aging. Notably, less than half of these benefits are attributable to improvements in classical risk factors (blood pressure, insulin sensitivity, lipid profile, body composition) [148]; the remainder likely reflects direct effects on the vascular wall, including enhanced nitric oxide (NO) bioavailability and structural remodeling [149].

Epidemiological evidence supports this view: higher cardiorespiratory fitness correlates with improved endothelial performance, while longer exercise duration is inversely related to arterial stiffness and wall thickness [150,151]. Longitudinal data show that lifelong aerobic exercise markedly attenuates the age-related decline in endothelial function [152]. While endothelial responsiveness improves within weeks of training, structural changes in vascular stiffness often require longer or may remain modest [153]. Benefits are consistent across sex and age groups [154], and are evident even in children and adolescents, as shown in the European Youth Heart Study [155]. These findings highlight the need to target preventive strategies early, particularly given the global rise in obesity and sedentary behavior [156]. Importantly, vascular responsiveness to exercise persists even into advanced age [157].

Mechanistic insights further reinforce the role of exercise as mitochondrial therapy. Park et al. demonstrated that chronic aerobic training enhances vascular mitochondrial respiratory efficiency without increasing mitochondrial content. In murine models, exercise increased state 3 respiration and respiratory control ratio, reduced mROS, and upregulated PGC-1α, MnSOD, and phosphorylated eNOS, thereby improving endothelial function [158]. These adaptations underscore mitochondrial functional plasticity as a key determinant of vascular health and support endurance exercise as a non-pharmacological strategy to counter endothelial dysfunction and age-related vascular decline.

## 6. Conclusions

The growing recognition of mitochondria as master regulators of vascular cell fate has reframed atherosclerosis—not as a simple disorder of lipid deposition, but as a progressive mitochondrial disease of the arterial wall. By integrating metabolic dysfunction, mROS-driven signaling, defective mitophagy, DAMPs-mediated inflammation, and maladaptive vascular remodeling, dysfunctional mitochondria emerge as the unifying mechanism underlying endothelial dysfunction, VSMC phenotypic switching, and macrophage-driven plaque instability.

This convergence positions mitochondrial reprogramming not as a peripheral adjunct, but as a paradigm-shifting strategy with the potential to reshape cardiovascular therapy. Novel interventions—ranging from modulation of the SIRT1/PGC-1α axis to mitochondria-targeted antioxidants, peptides, and nanocarriers—offer unprecedented opportunities to restore cellular energetics and redox balance. When complemented by omics-based diagnostics and advanced molecular imaging, these tools herald a future of personalized, organelle-centric medicine.

Importantly, lifestyle interventions such as endurance exercise act as physiological mitochondrial reprogrammers, reinforcing the synergistic potential of combining behavioral and molecular approaches. The concept of “targeting the mitochondrion” thus transcends traditional cardiovascular paradigms, providing a unifying mechanistic framework to address residual risk and enhance plaque stability.

In light of the multidimensional role of mitochondrial dysfunction in AS, we propose that vascular mitochondrial reprogramming should be regarded not as an ancillary option, but as a central pillar of next-generation strategies for AS prevention and treatment.

## Data Availability

No new data were created or analyzed in this study.

## Abbreviations

The following abbreviations are used in this manuscript:

CVDs	Cardiovascular diseases
AS	Atherosclerosis
ATP	Adenosine triphosphate
mROS	Mitochondrial reactive oxygen species
mtDNA	Mitochondrial DNA
OXPHOS	Oxidative phosphorylation
ETC	Electron transport chain
VSMCs	Vascular smooth muscle cells
MitoQ	Mitoquinone
AMPK	AMP-activated protein kinase
